# Development of Highly Multiplex Targeted Proteomics Assays in Biofluids Using a Nominal Mass Ion Trap Mass Spectrometer

**DOI:** 10.1016/j.mcpro.2026.101506

**Published:** 2026-01-07

**Authors:** Deanna L. Plubell, Philip M. Remes, Christine C. Wu, Cristina C. Jacob, Gennifer E. Merrihew, Chris Hsu, Nick Shulman, Brendan X. MacLean, Lilian Heil, Kathleen L. Poston, Thomas J. Montine, Michael J. MacCoss

**Affiliations:** 1 Department of Genome Sciences, University of Washington, Seattle, Washington, USA; 2 Thermo Fisher Scientific, San Jose, California, USA; 3 Department of Neurology & Neurological Sciences, Stanford University, Palo Alto, California, USA; 4 Department of Pathology, Stanford University, Palo Alto, California, USA

**Keywords:** targeted proteomics, quantitative analysis, neurodegenerative disease, plasma, cerebrospinal fluid

## Abstract

The development of targeted assays that monitor biomedically relevant proteins is an important step in bridging discovery experiments to large scale clinical studies. Targeted assays are currently unable to scale to hundreds or thousands of targets. We demonstrate the generation of large-scale assays using a novel hybrid nominal mass instrument. The scale of these assays is achievable with the Stellar mass spectrometer through the accommodation of shifting retention times by real-time alignment, while being sensitive and fast enough to handle many concurrent targets. Assays were constructed using precursor information from gas-phase fractionation data-independent acquisition (DIA). We demonstrate the ability to schedule methods from orbitrap and linear ion trap acquired gas-phase fractionation DIA library, and compare the quantification of a matrix-matched calibration curve from orbitrap DIA and linear ion trap parallel reaction monitoring (PRM). Two applications of these proposed workflows are shown with a cerebrospinal fluid neurodegenerative disease protein PRM assay and with a Mag-Net enriched plasma extracellular vesicle protein survey PRM assay. In cerebrospinal fluid, our assay targets proteins discovered previously to be associated with Alzheimer’s disease in a small independent sample set. For the Mag-Net enriched plasma survey assay, we observe that proteins selected based on their measurement robustness are still able to capture differences in abundance across disease groups in a small sample set. These highlight the application of highly multiplex, targeted protein assays in clinical research.

## Highlights

• Gas phase fractionation libraries enable rapid development of targeted proteomics assays.
• The Stellar MS can be used to target >2000 peptides in a single injection.
• A nominal mass ion trap mass spectrometer offers excellent quantitative performance.
• Targeted assays detect known Alzheimer's- and Parkinson's-associated proteins.
• Survey-style PRM assays capture disease biology without prior hypotheses.

## Graphical Abstract

**Figure:**
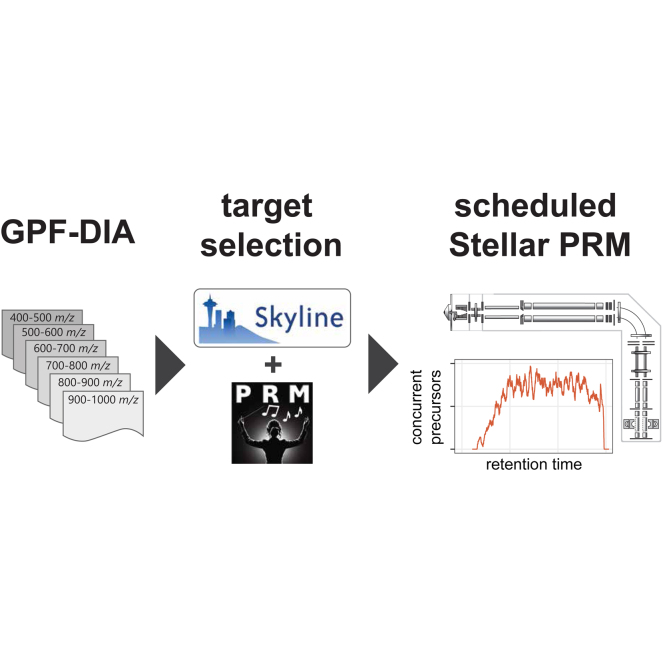

## In Brief

Targeted proteomics assays are essential for translating biomarker discoveries into clinical applications, but current methods struggle to scale beyond a few hundred targets. This study demonstrates that the Stellar mass spectrometer, combined with gas-phase fractionated DIA libraries and real-time retention time alignment, enables highly multiplex PRM assays measuring over 2000 peptides in a single injection. Applied to cerebrospinal fluid and plasma extracellular vesicles, these assays successfully quantify neurodegenerative disease-associated proteins, bridging discovery proteomics and large-scale clinical studies.

An important lesson from the last decade is that immunoassays, or any other assay that relies on affinity reagents, are far from a perfect solution to the protein quantification problem. While the use of immunoassays has historically been viewed as the necessary endpoint for putting a protein assay into clinical practice, the negatives are beginning to outweigh the strengths. Multiplex affinity assays have been widely used in applications of quantitative protein measurements across 1000s of plasma or serum samples. While increased multiplexing to 100s or 1000s of proteins can minimize sampling bias and provide a more comprehensive sampling of the proteome, it can present significant challenges. Despite the promised depth of coverage of low abundance proteins in both SOMAScan and Olink, LC-MS is still the gold standard method for providing selective protein measurements (1, 2, 3). Mass spectrometry has always outperformed affinity-based methods when specificity is the priority (4, 5, 6). In addition, mass spectrometry’s ability to measure multiple peptides per protein provides an opportunity to capture signals from different proteoforms (7).

Because of limitations of affinity-based protein assays, targeted mass spectrometry assays were viewed as a promising alternative to immunoassays in the clinical lab. Early methods for performing targeted protein quantitation were performed using an acquisition strategy known as selected reaction monitoring (SRM) on a triple quadrupole mass spectrometer (8, 9, 10, 11, 12). This method was powerful since this hardware was common in mass spectrometry labs focused on development of targeted assays. More recently, parallel reaction monitoring (PRM) has become a promising alternative to SRM. In PRM, the entire product ion spectrum is measured, which enables the confirmation of the identity of an analyte, and the refinement of product ion transitions post-acquisition to remove those with interference (13, 14, 15). Additionally, PRM only requires the selection of a precursor *m/z* and charge for monitoring, simplifying the development of these assays compared to SRM which requires selection of specific transitions to target.

Despite the promise of targeted proteomics, there have been limitations in the number of peptides that can be measured due to constraints on the number of concurrent analytes that can be measured at any point in time. As the number of concurrent precursors increases, the time spent on each target decreases, sacrificing the sensitivity and precision of the measurement. In an effort to increase the number of targets that can be measured, data-independent acquisition (DIA) has emerged as an alternative strategy where relatively wide MS/MS isolation windows are acquired spanning a predefined mass range (16, 17, 18, 19, 20). This acquisition results in a decrease in the number of scan events necessary per cycle. However, this intentional multiplexing of the MS/MS acquisition results in spectra that are highly chimeric and comes at the expense of selectivity and dynamic range.

Another way to increase the number of peptide targets is to incorporate retention time scheduling into LC-MS/MS acquisition method (21, 22, 23). It is common to monitor each target for 2 to 5 min, despite peptide peak widths of <20 s. This inefficient scheduling is necessary because of chromatographic shifts that occur over the lifespan of an HPLC column. Irreproducible retention time is particularly pronounced in the nanoliter to microliter per minute flow rates. Recently we reported a prototype data acquisition strategy where the instrument automatically assessed the retention time relative to a reference analysis and adjusted the instrument target list to only sample a peptide in the time immediately prior and after the peptide was expected (22). This was accomplished while accommodating non-linear and irregular shifts in retention time.

While the power of targeted assays is undeniable, the development of robust methodology for the selection of peptides to use as a proxy of a translated gene product is not straightforward. Due to differences in their inherent physicochemical properties, equimolar peptides of different amino acid sequences can have drastically different responses in a mass spectrometer. This development often relies on the use of stochastically sampled protein digests using data dependent acquisition (DDA) to determine which peptides should be targeted. This strategy is predicated on the assumption that the peptides most frequently identified in DDA experiments will produce peptides with the optimal signal-to-noise ratios for a targeted proteomic experiment – unfortunately, this is not the case (24, 25). There are numerous reasons why a peptide may not be selected for MS/MS during a DDA experiment. Therefore, a peptide that is not observed in this type of experiment should not be excluded in a targeted experiment. Conversely, a peptide that is routinely sampled in a DDA style experiment might not necessarily be a suitable peptide in a targeted analysis. Further challenges exist, such as determining the elution time for a sample matrix and a chromatographic setup and determining which precursor-to-product ion transitions should be monitored by SRM acquisition and analysis, or for PRM data analysis.

Our lab has shown that the use of gas-phase fractionation (GPF) with DIA-MS and narrow precursor isolation windows is a powerful strategy for assessing which peptides can be measured well in a DIA experiment with less specific precursor isolation (20, 26). More recently, we have shown that these DIA based chromatogram libraries are powerful for providing information about 1) which peptides are detectable, 2) the relative abundance of the fragment ions, 3) the retention time and chromatographic characteristics of each peptide, and 4) peptide stability – significantly expediting the targeted assay development process. This original strategy was used to streamline the development of SRM assays but this process is also relevant for PRM (27).

Here we describe the application of the Thermo Fisher Scientific Stellar mass spectrometer for the implementation of highly multiplex targeted assays in both human cerebrospinal fluid (CSF) and plasma. The Stellar instrument hardware is a combination of a quadrupole mass filter, an ion routing multiple, and a dual pressure, radial ejection, linear ion trap (LIT) (28). This hardware is conceptually similar to the Tribrid series instruments without the Orbitrap analyzer. Additionally, as with the Tribrid instruments, the Stellar can pipeline the quadrupole isolation and analyte fragmentation in parallel with the spectrum acquisition in the LIT. A unique capability of the Stellar is the implementation of an intelligent data acquisition strategy that can accommodate chromatographic retention time shifts by updating the scheduled target list using a real-time retention time alignment as described previously (22), now called Adaptive RT. Furthermore, the workflow for developing targeted PRM assays using GFP DIA data has been improved with a Skyline external tool called PRM Conductor (28).

## Experimental Procedures

### Experimental Design and Statistical Rationale

Both lumbar CSF and plasma in EDTA anticoagulant were collected for age and sex matched samples from four diagnosis categories: Parkinson’s disease cognitively normal (CSF n = 10, plasma n = 10), Parkinson’s disease with dementia (PDD; CSF n = 8, plasma n = 10), Alzheimer’s disease dementia (CSF n = 9, plasma n = 10), and healthy cognitively normal (CSF n = 10, plasma n = 10) as controls. Diagnostic criteria for Alzheimer’s disease dementia and Parkinson’s disease and the assessments for the cognitive status determination have previously been described (29). This study was conducted in accordance with international ethical standards and approved by the Human Subjects Institutional Review Board of Stanford University. Written informed consent was obtained from all participants or their legally authorized representative.

Matrix-matched calibration curves were made for both CSF and Mag-Net enriched plasma extracellular vesicle (EV) samples. For the CSF curve, a pool of human CSF was diluted into a diluted 10% chicken serum sample. For the Mag-Net enriched plasma EV samples, a pool of human plasma was diluted into chicken plasma (Innovative Research, Novi, MI). For both the CSF and Mag-Net enriched plasma EV samples, a calibration curve was made using volumetric human:chicken percentages or 100%, 70%, 50%, 30%, 10%, 7%, 5%, 3%, 1%, 0.7%, 0.5%, 0.3%, and 0.1%. Peptide sequences shared between chicken and human canonical fasta databases were removed for all figures of merit analysis.

### Cerebrospinal Fluid Protein Digestion

A 25-μg aliquot of each human CSF sample was diluted to 1% SDS, 50 mM Tris pH 8.5, containing 400 ng yeast enolase protein added as a process control. The sample was reduced with 10 mM TCEP and the free thiols alkylated with 15 mM iodoacetamide in the dark for 30 min. The remaining iodoacetamide was quenched with the addition of 10 mM DTT for 15 min. Proteins were prepared using protein aggregation capture (PAC) and trypsin digestion as described previously (30, 31, 32). Briefly, 130 μg of MagResyn Hydroxyl beads (ReSyn Biosciences) were added to the reduced and alkylated CSF sample for a 1:5.2 protein:bead ratio. The proteins were aggregated to the beads with the addition of 70% acetonitrile for 10 min. The beads were washed three times in 95% acetonitrile and 2 times in 70% ethanol for 2.5 min each using a Thermo Fisher Scientific KingFisher Flex. Peptides were released from the beads by adding the beads to 100 mM ammonium bicarbonate with trypsin (enzyme substrate ratio 1:20). The digestion was quenched by acidification to 0.5% trifluoroacetic acid and spiked with Pierce Retention Time Calibrant peptides to a final concentration of 50 fmol/μl.

### Plasma Extracellular Vesicle Enrichment and Digest Using Mag-Net

EVs were enriched from plasma samples and digested using a Thermo Fisher Scientific KingFisher Flex as described previously (32). Briefly, 100 μl of plasma was combined 1:1 with Binding Buffer (100 mM Bis-Tris Propane, pH 6.3, 150 mM NaCl). MagResyn strong anion exchange (SAX, ReSyn Biosciences) were first equilibrated 2 times with 500 μl Equilibration/Wash Buffer (50 mM Bis-Tris Propane, pH 6.5, 150 mM NaCl) with gentle agitation and then combined in a 1:4 ratio (volume beads:volume starting plasma) with the plasma:binding buffer sample for 45 min at room temperature. The beads were washed with 500 μl Equilibration/Wash Buffer three times for 5 min with gentle agitation. The enriched membrane particles on the beads were then solubilized and reduced in 100 μl 50 mM Tris, pH 8.5/1% SDS/10 mM Tris (2-carboxyethyl) phosphine (TCEP) at 37 °C and gentle agitation with 800 ng enolase standard added as an internal quality control (33). Following reduction, the plate was removed from the Kingfisher Flex. Samples were alkylated with 15 mM iodoacetamide in the dark for 30 min and then quenched with 10 mM DTT for 15 min. The samples were processed using PAC with minor modifications (30, 31, 32). Briefly, the samples were adjusted to 70% acetonitrile, mixed, and then incubated for 10 min at room temperature to aggregate proteins onto the SAX bead surface. The SAX beads were washed three times in 95% acetonitrile and 2 times in 70% ethanol for 2.5 min each. Samples were digested for 1 h at 47 °C in 50 mM Tris, pH 8.5 with Thermo Scientific Pierce porcine trypsin at a ratio of 20:1 protein to trypsin. The digestion was quenched in 0.5% trifluoroacetic acid and spiked with Pierce Retention Time Calibrant peptide cocktail (Thermo Fisher Scientific) to a final concentration of 50 fmol/μl. Peptide digests were stored at −80 °C.

### CSF Survey PRM Assays and Corresponding DIA

GPF DIA libraries were acquired both on an Exploris 480 Orbitrap and Stellar mass spectrometer linear ion trap. CSF peptide digest was separated with a 30-min gradient from Mobile phase A of 5% (0.1% Formic Acid in water) to 55% Mobile phase B (1% Formic acid in 80% Acetonitrile) on a 0.5 cm, 300 μm inner diameter trap column with a 15 cm 150 um inner diameter C18 Pepsep separations column with a Vanquish Neo UHPLC. Orbitrap GPF DIA data was acquired with an Exploris 480 instrument using 4 *m/z* staggered windows from precursor mass range 400 to 1000 *m/z*. Separate injections were performed to measure each 100 *m/z* precursor mass range (400–500 *m/z*, 500–600 *m/z*, 600–700 *m/z*, 700–800 *m/z*, 800–900 *m/z*, 900–1000 *m/z*), with normalized collision energy (NCE) of 27, 1000% normalized automatic gain control (AGC) target, and an orbitrap resolution of 30,000. Wider isolation window DIA was performed with an Exploris 480 for the 400 to 1000 *m/z* precursor mass range, with 12 *m/z* staggered windows with NCE of 27, 1000% normalized AGC target, and an orbitrap resolution of 30,000. Raw files were demultiplexed and converted to mzML with MSConvert v3.0.20070 and searched with EncyclopeDIA v2.12.30 with a Prosit library generated from a uniprot canonical human fasta (09/2022) with APOE isoforms and yeast enolase protein sequence added. Exploris 12 *m/z* DIA matrix-matched data were subsequently with EncyclopeDIA and the GPF chromatogram library (20, 26). All searches were performed using a uniprot canonical human fasta (09/2022) with APOE-ε2 and APOE-ε4 isoform peptides and yeast enolase protein sequence (P00924) added as the background proteome, with normal target/decoy, trypsin enzyme, CID/HCD fragmentation, 10 ppm precursor, fragment, and library mass tolerance, using Percolator v3.01, and a minimum of three transitions with no interference. Data for precursors passing 1% FDR were extracted in Skyline-Daily v23.1.1.503 where protein parsimony was performed and non-unique peptides removed.

Linear ion trap GPF DIA data was acquired with a Stellar mass spectrometer using 2 *m/z* discrete windows with optimized placement for the precursor mass range 400 to 1000 *m/z*, with NCE of 30%, 125 kDa/s scan rate, dynamic AGC target of 1.0e4, and automatic maximum injection time. Stellar MS-DIA data was searched with MSFragger v3.8 and post-processed with Percolator v3.05 within Skyline. The data was searched against the Uniprot canonical human fasta (09/2022) with APOE isoforms and yeast enolase protein sequence added. MSFragger was searched using a precursor tolerance of ± 1 Da and a fragment tolerance of 0.2 Da. Data for precursors passing 1% FDR were extracted in Skyline where protein parsimony was performed and non-unique peptides removed.

Two assays were generated from the peptide information in either the Exploris 480 Orbitrap acquired GPF library or the Stellar MS linear ion trap GPF library. CSF peptide digest was separated on a 0.5 cm, 300 um inner diameter trap column with a 15 cm 150 μm inner diameter C18 PepSep separations column with a Vanquish Neo UHPLC. PRM methods were constructed with an Adaptive RT experiment, a full MS1 acquisition, and a triggered MS2 acquisition with the target list and tentative retention time window schedule. The retention time alignment MS scan was 350 to 1250 *m/z* at 200 kDa/s scan rate, 0.01 s per alignment cycle, AGC target of 3.0e4, and an automatic maximum injection time. The MS1 scan was for 200 to 1500 *m/z*, with an AGC target of 3.0e4. The targeted MS2 were performed with 1 *m/z* isolation windows, NCE of 30%, 125 kDa/s scan rate, RF lens of 30%, dynamic AGC target of 1.0e4, dynamic maximum injection time, 2.15 s cycle time for an estimated six points per peak (avg peak width ∼13.9 s), and Adaptive RT dynamic time scheduling using the.rtbin file created by PRM conductor. Precursor targets for each PRM assay were selected with the PRM conductor tool in Skyline.

For the assay generated from the Exploris orbitrap 480 GPF DIA data a set of precursors were selected by filtering for precursors with a minimum absolute area of 1000, a minimum and maximum peak width of 5.2 and 26.1 s, and a minimum of three interference-free transitions. To test the transfer of the alignment between the Exploris and Stellar MS setups a preliminary set of scheduling runs were performed on the Stellar MS ion trap with a 125 kDa/s scan rate, MS2 scan range of 200 to 1500 *m/z*, a target of six points across the peak for 13.07 average measured peak widths resulting in 2.18 s cycle times with optimized window widths and a minimum of 2.4 min windows. Data was extracted and inspected in Skyline, with some manual peak re-integration to match the library dot product and expected relative retention time. A PRM assay with narrow 0.75 min windows was scheduled based on the 2.4 min precursor window PRM data, with a scan rate of 125 kDa/s, minimum target injection time of 15 ms, scan range from 200 to 1500 *m/z*, expected LC width of 12.9 s, and a minimum of six points per peak resulting in a 2.15 s cycle time with optimized window widths and a minimum of 0.75 min windows. The OT GPF derived PRM assay was reduced from 3587 precursors from 1735 proteins in the preliminary scheduling runs to 2367 precursors from 1486 proteins for the final assay. This was done to reach comparable cycle times and total number of precursors targeted in the assay generated from the Stellar GPF DIA.

For the PRM assay generated from the Stellar MS linear ion trap GPF DIA data a set of precursors were selected by filtering for precursors with a minimum absolute area of 500, a minimum and maximum peak width of 5.2 and 45 s, and a minimum of three interference-free transitions. A scheduled PRM assay was generated with a scan rate of 125 kDa/s, minimum target injection time of 15 msec, scan range from 200 to 1500 *m/z*, expected LC width of 12.93 s, and a minimum of six points per peak resulting in a 2.15 s cycle time with optimized window widths and a minimum of 0.75 min windows.

For the matrix-matched calibration curves an initial acquisition of each PRM assay was performed on a 50% human CSF in chicken serum to help schedule the appropriate retention time alignment with lower concentrations of human CSF in chicken serum. Alignment of the peaks picked in the 50% were manually inspected to ensure accurate peak picking to match the library spectra. After filtering the LIT GPF derived PRM assay was reduced from 2347 precursors from 917 proteins to 2248 precursors from 876 proteins for the final assay. Each CSF dilution (100%, 70%, 50%, 30%, 10%, 5%, 1%, 0.5%, and 0.1%) was acquired in triplicate for both PRM assays, with blank injections between each curve.

### CSF Neurodegenerative Disease Protein PRM Assay

Peptides were separated on a 15 cm, 75 μID C18 IonOpticks column by a Vanquish Neo UHPLC and analyzed by DIA and PRM with a Stellar mass spectrometer. A GPF library was collected using 2 Th windows for the precursor mass range 300 to 1100, with each injection spanning 100 *m/z* of the precursor mass range, with NCE of 30%, 125 kDa/s scan rate, dynamic AGC target of 1.0e4, and automatic maximum injection time. GPF DIA was searched with Proteome Discoverer software with Chimerys search algorithm using the inferys_3.0.0_fragmentation prediction model, with parameters for peptides of 7 to 30 amino acids with charge +2 to +4, up to 2 missed cleavages, 0.7 Da fragment tolerance, dynamic oxidized methionine modification with a maximum of 2 modifications per peptide, searched with a uniprot canonical human fasta (09/2022) with APOE isoforms and yeast enolase protein sequence added. A list of 105 proteins of interest was compiled from previous studies, with 101 proteins with detectable peptides in the 60m LIT GPF DIA library. For these 101 proteins, peptides with missed cleavages were removed (except for the PRDX2 protein, where the missed cleavage peptides were the only unique peptides).

PRM conductor was used to generate a scheduled assay for 1070 precursors after filtering for peaks widths with a minimum of 6 s and a minimum of three transitions with no interference. PRM methods were constructed with an Adaptive RT DIA experiment with twelve 50 Th isolation width acquisitions, followed by a full MS1 acquisition, and a targeted MS2 experiment with the target list. The retention time alignment DIA experiment measured 400 to 1000 *m/z* with 200 kDa/s scan rate, 0.01 s per alignment cycle, AGC target of 3.0e4, and an automatic maximum injection time. The MS1 scan was for 200 to 1500 *m/z*, with an AGC target of 3.0e4. The targeted MS2 were performed with 1 *m/z* precursor isolation windows, NCE of 30%, 125 kDa/s scan rate, RF lens of 30%, AGC target of 1.0e4, dynamic maximum injection time, 1.71 s cycle time for a estimated seven points per peak (avg peak width ∼12 s), and Adaptive RT dynamic time scheduling using the.rtbin file created by PRM conductor. Precursor targets for each PRM assay were selected with the PRM conductor tool in Skyline (1.5 min windows with optimized widths). For the individual dementia CSF samples peaks were manually inspected, and peptides with truncated peaks in >20% of the samples removed from further analysis.

### Mag-Net Enriched Plasma extracellular vesicle Survey PRM Assays of Different Sizes

One μg of digested sample was loaded onto a μPAC HPLC column with a 75 μm internal diameter and 50 cm length. The separation was performed using a flow rate of 350 nl/min using a Vanquish Neo HPLC. Mobile phase A was 0.1% formic acid in water, and mobile phase B was 80% acetonitrile and 0.1% formic acid in water. The gradient ran from 0% B to 30% B in 22.5 min, increased to 45% B at 30 min, increased again to 99% B at 30.25 min, and remained at 99% B until 35.25 min. Stellar Linear ion trap GPF DIA data was acquired with a Stellar mass spectrometer using 1 *m/z* discrete windows with optimized placement for the precursor mass range 400 to 1000 *m/z*. GPF DIA data were searched with Proteome Discoverer software with SEQUEST and Inferys rescoring, and precursors passing 1% FDR used for further analysis.

Two separate PRM assays were constructed: a “large” assay targeting 3501 precursors and a “small” assay targeting 1599 precursors. Precursor targets for the larger assay were selected from the Stellar LIT GPF DIA using PRM conductor, filtering for precursors with a minimum absolute area of 500, a minimum and maximum peak width of 4 and 17 s, and a minimum of three interference-free transitions. Precursors in the small PRM assay were selected by filtering targets from the 3501-assay based on their performance in the matrix-matched calibration curve. Specifically, targets were selected for the small assay if they had <20% CV and were within 50% of the expected ratio. PRM methods were constructed with an Adaptive RT DIA experiment with twelve 50 Th isolation width acquisitions, followed by a full MS1 acquisition, and a targeted MS2 experiment with the target list. The retention time alignment DIA experiment measured 400 to 1000 *m/z* with 200 kDa/s scan rate, 0.01 s per alignment cycle, AGC target of 3.0e4, and an automatic maximum injection time. The MS1 scan was for 200 to 1500 *m/z*, with an AGC target of 3.0e4. The targeted MS2 was performed with 1 *m/z* precursor isolation windows, NCE of 30%, 125 kDa/s scan rate, RF lens of 30%, and dynamic AGC target of 1.0e4, dynamic maximum injection time, and Adaptive RT dynamic time scheduling using the.rtbin file created by PRM conductor.

### Mag-Net Enriched Plasma EV Survey PRM Assay for a Dementia Cohort

One μg of a digested pooled human enriched plasma EV sample was loaded onto a μPAC HPLC column with a 75 μm internal diameter and 50 cm length and separated with a 30 min gradient from Mobile phase A of 5% (0.1% Formic Acid in water) to 55% Mobile phase B (1% Formic acid in 80% Acetonitrile) using a Vanquish Neo HPLC. Stellar linear ion trap GPF DIA data was acquired with 1 *m/z* discrete windows with optimized placement for the precursor mass range 400 to 1000 *m/z.* Stellar GPF DIA data were searched with Proteome Discoverer software with SEQUEST and Inferys rescoring. Existing results from an Eclipse GPF DIA experiment acquired with 4 *m/z* staggered windows and searched with EncyclopeDIA and a Prosit predicted library, were combined with the Stellar GPF DIA results, and a reduced fasta file generated. The Stellar GPF DIA was then searched against the reduced fasta, and results passing a 1% FDR extracted in Skyline.

Precursor targets were selected with PRM Conductor, filtering for a minimum absolute area of 300, a minimum and maximum peak width of 5 and 17 s, and a minimum of 3 coeluting transitions without interference. A PRM assay was scheduled with 0.8-min windows and 1.43 s cycle time, resulting in three separate injections to cover 4136 precursors. The assay was acquired in triplicate with the Stellar MS PRM method with an Adaptive RT DIA experiment with twelve 50 Th isolation width acquisitions, followed by a full MS1 acquisition, and a targeted MS2 experiment with the target list. Results from this preliminary assay were used to further filter for precursors with a total ion current-normalized % CV below 30% across the three replicates. The final assay targeting 2093 precursors with a 1.5 s cycle time was scheduled through PRM Conductor and used to measure all individual dementia plasma EV samples.

### Statistical Analysis

PRM and DIA data were extracted in Skyline Daily with their corresponding libraries. For the matrix matched calibration curves the peak integration boundaries were determined with a python script that uses boundaries from the 100% samples to calculate boundaries for the more dilute samples. The limit of detection and limit of quantification is determined with a python script based on previously described methods (34), with an additional step to select optimal transitions for improving the figures of merit (35). Additional statistical analysis and generation of figures was performed in R v4.3.1. Differential abundance testing significance is determined by a Benjamini-Hochberg adjusted *p*-value of 0.05%. All coefficients of variation calculations are performed on non-log normalized data.

## Results

### Scheduling LIT PRM Assays with Adaptive Real-Time Alignment

The Stellar mass spectrometer was configured to collect PRM data using its Adaptive RT algorithm. The Adaptive RT algorithm has been described in detail previously (22). In brief, reference spectra from DIA spectra with 50 *m/z* wide isolation windows can be compressed and serialized to a file having the extension.rtbin. This file can be selected and embedded into targeted MSn instrument methods, which causes the method editor to include those same DIA acquisitions into the assay. During execution of the targeted MSn assay, the data from the DIA acquisitions are compared with the embedded data in real-time to estimate retention time shifts compared to the reference and to update the set of active targets at a period of 5 times per LC peak width. Creation of the.rtbin file and embedding into a method can be automated with the PRM Conductor program.

### A Generalized Workflow for the Development of Reproducible, Highly Multiplex Protein PRM Assays

**Fig. 1:**
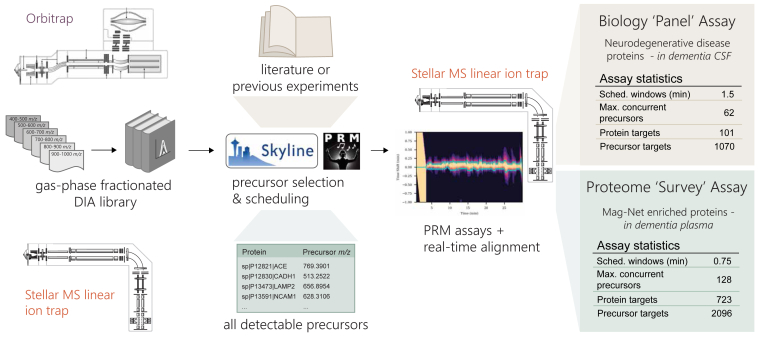
**Workflow for the generation of scheduled PRM assays with GPF DIA libraries.** GPF-DIA libraries can be acquired either directly on a Stellar MS linear ion trap or by an Orbitrap instrument, and their results are extracted in Skyline. With the use of the PRM conductor tool in Skyline precursors can be refined for chromatographic performance and signal, and precursors can be selected to optimize both the selection of precursor *m/z* and relative retention time. Selected precursors are then acquired by PRM on the Stellar MS with dynamic real-time alignment. We demonstrate the selection of precursors for an assay targeting a panel of neurodegenerative associated proteins in CSF, and the selection of precursors for a ‘survey’ assay of the measurable proteome of Mag-Net enriched plasma. CSF, cerebrospinal fluid; DIA, data-independent acquisition; GPF, gas-phase fractionation; PRM, parallel reaction monitoring.

Previous work from our group has demonstrated the use of GPF DIA for the assessment of the peptides and proteins detectable in a given sample matrix (20, 26). The information acquired in these libraries can then be used to inform the selection of precursors for targeted assays (27). We use GPF-DIA libraries both acquired with the Orbitrap (OT) analyzer of an Exploris 480 mass spectrometer or with the LIT of a Stellar mass spectrometer to show the feasibility of generating assays that are selected from and aligned to DIA data from either an OT system or from the same Stellar MS LIT used for the PRM acquisition (Fig. 1). We generated two types of assays based on the GPF DIA data. A neurodegenerative disease protein assay was constructed to measure all detectable peptides derived from a set of proteins informed by both literature and previous experimental results in CSF. Another PRM assay was constructed for measuring the maximum reproducibly sampled precursors in Mag-Net enriched plasma EVs in dementia.

### Generation and Scheduling of PRM Assays Using Gas-phase Fractionation Libraries

**Fig. 2:**
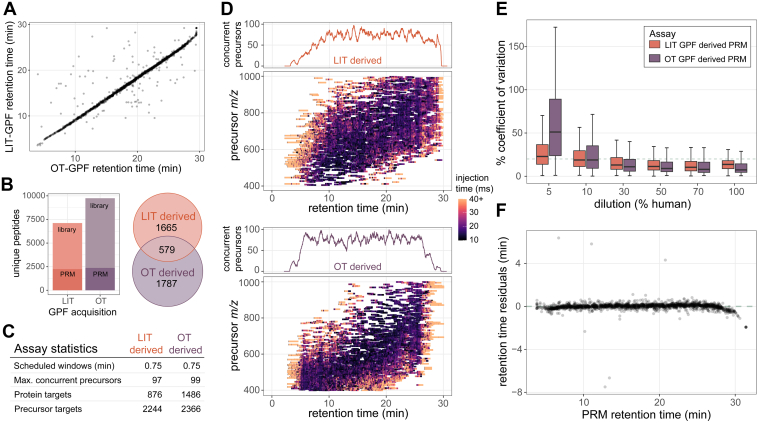
**CSF survey PRM assays developed from either Stellar MS linear ion trap (LIT) GPF library or Orbitrap (OT) GPF library data.** GPF DIA was acquired by Exploris Orbitrap 480 OT with 4 *m/z* staggered windows, or by Stellar MS LIT with 2 *m/z* discrete windows. *A*, the correlation of retention times for precursors detected in both the LIT and OT GPF DIA libraries. *B*, precursor targets were selected from both libraries for separate LIT PRM assays using the PRM Conductor tool to filter and maximize the number of precursors. *C*, general characteristics of the two assays generated from either GPF DIA library. *D*, the number of concurrent precursors across the gradient for each assay, and the targeted precursor acquisition windows from PRM conductor across the precursor mass range and retention time space for both assays. Individual MS2 scans are plotted and colored by the ion injection time. *E*, the % CV from peptides targeted in both assays at 5%, 10%, 50%, and 100% human CSF dilutions in chicken serum. *F*, residuals from predicted retention times based on the OT GPF library and the measured retention times on the Stellar PRM assay derived from the OT GPF library. LIT, linear ion trap; CSF, cerebrospinal fluid; DIA, data-independent acquisition; GPF, gas-phase fractionation; PRM, parallel reaction monitoring; OT, Orbitrap.

The GPF library acquired with 4 *m/z* overlapping windows from the OT did result in a larger number of peptides and proteins detected when compared to the LIT library searched with MSFragger. For the precursors that were detected in both systems there is a strong correlation in the observed retention times (Fig. 2*A*). From these two libraries two separate assays were constructed to measure as many precursors or proteins as possible while maintaining an average of six points across the peak for 13.9 s peak average. For the assay generated from the LIT GPF a total of 2248 precursors mapping to 876 proteins were targeted, while the assay generated from the OT GPF targeted a total of 2367 precursors mapping to 1486 proteins. Both assays targeted a different portion of the proteome, with the assay generated from the LIT GPF library prioritizing the number of precursors and the assay generated from the OT GPF library prioritizing the number of proteins, while also maintaining a similar number of precursors (Fig. 2, *B* and *C*).

The precursors selected from the LIT GPF are distributed evenly both across chromatographic separation and precursor *m/z*. The precursors selected from the OT GPF were also distributed evenly across retention time, while enriched for precursors in the lower precursor *m/z* range (Fig. 2*D*). For precursors targeted in both PRM assays the OT GPF based assay has slightly lower median %CV for the least dilute points on a matrix-matched calibration curve (Fig. 2*E*). At around 10% human CSF in the chicken serum background, both assays have similar medians, while lower concentrations have worse reproducibility in the OT GPF based assay. PRM acquired on the Stellar MS system has a strong correlation in retention times to the OT GPF library detections with the same separation conditions (Fig. 2*F*). An initial assay consisting of 4 separate injections with 2 min windows were compared to the original OT GPF data, which ensured accurate alignment and scheduling for the 0.75 min scheduled single injection method.

### Highly Multiplex LIT PRM has Similar Quantitative Capacity in CSF as Orbitrap DIA

**Fig. 3:**
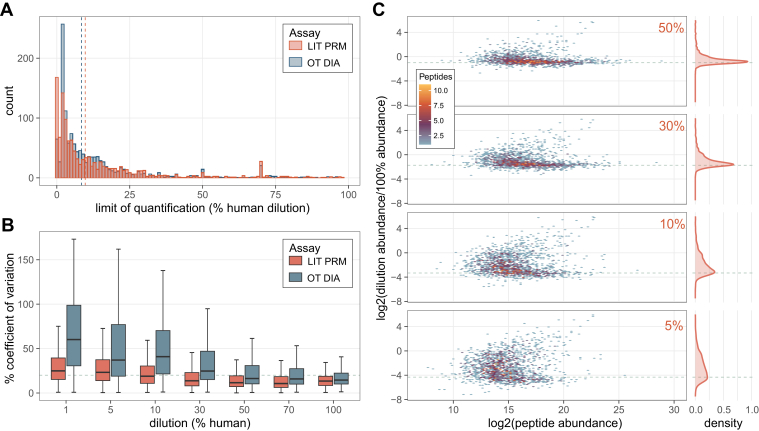
**Comparison of quantification by Stellar MS PRM and OT DIA.** Calibration curves of cerebrospinal fluid diluted into chicken serum were used to construct figures of merit. All data shown are for peptides measured on both Stellar MS LIT PRM assay and Exploris OT DIA experiment with 6 *m/z* precursor windows after demultiplexing. *A*, the distribution of the limit of quantification after transition optimization for both systems. *B*, CV from triplicate curve measurements. *C*, distribution of peptide ratios at points on the calibration curve acquired by Stellar MS LIT PRM, ratios are calculated as the log2 ratio of mean summed precursor area of the dilution to the 100%. *Dashed line*s indicate the expected ratios for each dilution point. DIA, data-independent acquisition; LIT, linear ion trap; PRM, parallel reaction monitoring.

To benchmark the quantitative performance of highly multiplex assays on the Stellar MS we compared a matrix-matched calibration curve of CSF into chicken serum acquired by 12 *m/z* staggered window DIA (6 *m/z* precursor specificity after demultiplexing) on the Exploris 480 OT and by PRM on the Stellar MS. The PRM assay was constructed for 2244 precursors detected in a LIT GPF library as described above. For peptides measured in both experiments there was a similar distribution in the calculated limits of quantitation, with the DIA median 8.5% and PRM median 9.7% (Fig. 3*A*). The reproducibility of the PRM was better at lower dilutions on the calibration curve compared to the same peptides measured by DIA. The accuracy of the quantification was assessed by calculating the log2 ratio of the diluted sample peptide abundance by the undiluted sample peptide abundance. The observed ratio distributions for the PRM assay are tightly centered around the expected ratios for the higher % human samples, with the distribution widening at lower concentrations (Fig. 3*C*).

### PRM Assay Size and Quantification in Mag-Net Enriched Plasma EV Particles

**Fig. 4:**
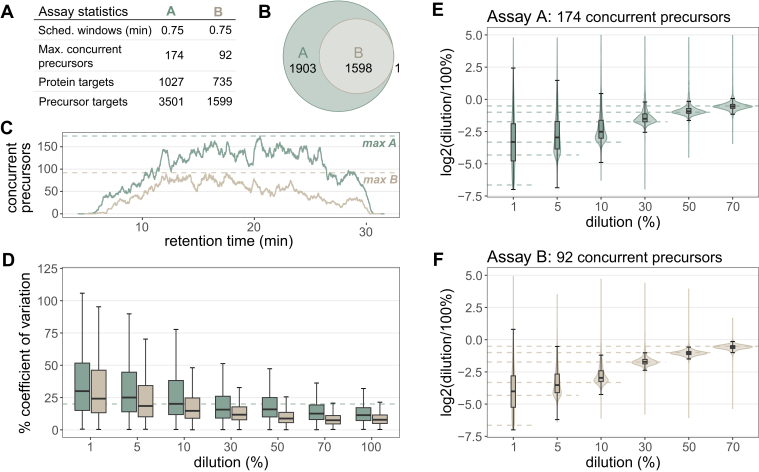
**Quantitative performance of Mag-Net enriched plasma EV PRM survey assay.***A*, A GPF DIA library was acquired on the Stellar mass spectrometer, and two separate assays were constructed for either 3501 or 1599 precursors. Assays were acquired on a 30 min gradient, with 0.75 scheduled windows. *B*, assay B was a subset of the larger Assay A, with more stringent precursor filtering parameters. *C*, the number of concurrent precursors for both Assay A and Assay B, with the maximum indicated by *dashed line*. Figures of merit were constructed from a matrix-matched calibration curve analyzed by both assays. *D*, the precision of the assays from 1% to 100% dilutions as measured by % CV across triplicate injections, with the *dashed line* indicating a 20% CV. The accuracy of the assay from 1% to 70% dilutions, calculated as the log2 ratio of mean summed precursor area of the dilution to the 100% for *E*) Assay A and *F*) Assay B. *Dashed lines* indicate the expected ratios for each dilution point. DIA, data-independent acquisition; GPF, gas-phase fractionation.

While the Stellar is capable of measuring a large number of peptide targets, we wanted to examine the quantitative figures of merit of these large assays. Two assays were constructed using PRM Conductor with a GPF DIA library with 1 *m/z* windows on Mag-Net enriched human plasma EVs. For the larger Assay A, 3501 precursors mapping to 1027 proteins were targeted with 0.75 min scheduled windows, resulting in a maximum of 174 concurrent precursors on a 30 min gradient. For the smaller Assay B 1599 precursors mapping to 735 proteins were targeted with 0.75 min scheduled windows, resulting in a maximum of 98 concurrent precursors on a 30-min gradient (Fig. 4*A*). Both assays were used to acquire a matrix-matched calibration curve in triplicate, constructed from digested Mag-Net enriched EVs prepared from human plasma diluted into chicken plasma. The larger of the two assays (A) was less precise across all dilution points, with the median % CV surpassing 20% at the 10% dilution in the large assay (20.2%) and at the 1% dilution in the smaller assay (24.4%) (Fig. 4*C*). The observed ratio distributions are tightly centered around the expected ratios for the higher % human samples for both assays, with the smaller Assay B distributions still closely centered around the expected ratio down to 5% human dilution (Fig. 4, *D* and *E*).

### Stellar MS PRM Assay Targeting Neurodegenerative Disease Proteins of Interest in CSF

**Fig. 5:**
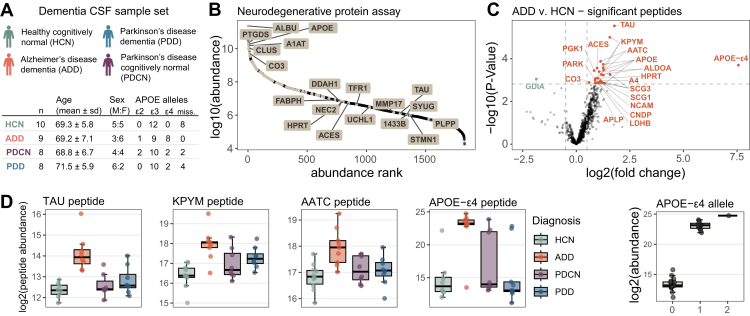
**CSF neurodegenerative disease associated protein PRM assay.***A*, dementia CSF sample set statistics. *B*, the distribution of the targeted proteins (*highlighted*) across the whole CSF dynamic range as determined by orbitrap GPF DIA library. *C*, differential abundance of target precursors between samples diagnosed with Alzheimer’s disease (AD) and healthy controls (HC). Precursors are labeled with their corresponding protein ID, and Benjamini-Hochberg adjusted *p*-value <0.05 colored, with *orange* representing precursors increased in AD relative to HC, and *green* decreased in AD relative to HC. *D*, select peptides with significantly different abundance in AD compared to the other diagnosis groups. *E*, APOE-ε4 isoform specific peptide LGADMEDVR abundance separated by the number of APOE-ε4 alleles based on genotyping. The abundance of the peptide with zero alleles is based on the integration of the background signal in the absence of the peptide. AD, Alzheimer’s disease; CSF, cerebrospinal fluid; GPF, gas-phase fractionation; HC, healthy control.

A list of 105 proteins was compiled from previously published targeted assays and discovery proteomics results for Alzheimer’s and Parkinson’s disease characterization (36, 37). From a 60 min, 2 *m/z* window GPF DIA library acquired on the Stellar mass spectrometer we detected peptides mapping to 101 of those proteins (Fig. 5*B*). After filtering for signal quality and setting a limit of 1.7 s cycle time to target approximately six spectra per peak we selected 1070 precursors with window optimization and a minimum of 1.5-min windows. This resulted in a neurodegenerative disease panel assay with 62 concurrent precursors and dynamic real-time alignment.

The neurodegenerative disease panel assay was used to measure a dementia CSF sample set consisting of individuals diagnosed as both Alzheimer’s disease dementia (AD) and Parkinson’s disease with and without dementia (PDD and Parkinson’s disease cognitively normal respectively) (Fig. 5*A*). Of the peptides measured 29, mapping to 18 proteins, were significantly different (adj. *p*-Value <0.05) between the AD and healthy cognitively normal individuals (Fig. 5*C*). The peptide mapping to Tau protein was most significantly changing in AD, which has been described previously (38). Despite the small cohort size, we find quantitative differences largely because this assay was based on proteins previously shown or hypothesized to be associated with neurodegenerative disease in CSF. Two examples of proteins we observe with differentially abundant peptides in AD are ALDOA and KPYM (Fig. 5*D*), which have been previously observed (39).

The peptide with the largest fold change difference observed was the APOE-ε4 isoform specific peptide LGADMEDVR. APOE-ε4 alleles are found at a higher frequency in individuals diagnosed with AD compared to the general population, making it a risk factor for disease (40, 41). Our sample set recapitulates this observation, with 7 AD individuals having 8 ε4 alleles (44% ε4 allele frequency), compared to 4 ε4 alleles in the rest of the individuals with genotyping data available (19 individuals; 10.5% ε4 allele frequency). When the APOE-ε4 peptide abundance is grouped by APOE-ε4 allele, the abundance tracks with the known genotype (Fig. 5*E*). The signal reported for the APOE-ε4 peptide in individuals without the allele is due to background signal at the expected retention time from those precursor-product ion transitions. The quantitation of this APOE-ε4 allelic peptide highlights the capability of monitoring single amino acid variants using a targeted MS method (42).

### Mag-Net Enriched Plasma EV PRM Survey Assay

**Fig. 6:**
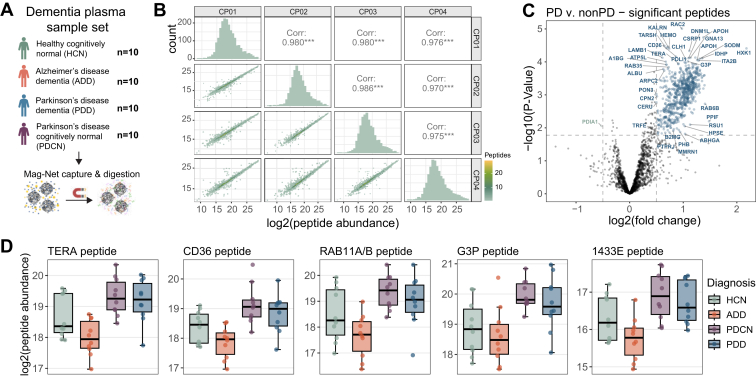
**Mag-Net enriched plasma EV survey PRM assay captures distinct differences in Parkinson’s disease.***A*, a plasma sample set consisting of age-matched healthy cognitively normal, Alzheimer’s disease dementia, Parkinson’s disease dementia, or Parkinson’s disease cognitively normal was enriched for EVs with the Mag-Net method and analyzed by PRM survey assay. *B*, peptide abundance correlations and distribution between sample preparations QC replicates. *C*, total ion current normalized peptide abundances between Parkinson’s with and without dementia compared to both healthy cognitively normal and Alzheimer’s disease dementia, with significantly changing peptides highlighted in *blue* if increased in Parkinson’s and *green* if increased in non-Parkinson’s samples. Peptides are labeled with their corresponding protein identifier. *D*, a selection of significantly changing peptides from proteins previously found to be differential in Parkinson’s disease studies. PRM, parallel reaction monitoring; EV, extracellular vesicle; PD, Parkinson’s disease.

For the Mag-Net enriched plasma EV particles, a targeted “survey style” assay was compiled for 2096 precursors mapping to 723 proteins. Precursors were selected and scheduled based on a GPF 1 *m/z* DIA library collected on a pool of the dementia plasma EV samples. We call the assay “survey style” because it was meant to represent a sampling of peptides that could be measured with high precision and accuracy and not necessarily provide classification between conditions or disease states. The reproducibility of peptide abundances from QC samples included in the experiment was reported previously (43) using an Orbitrap Eclipse with 12 *m/z* staggered window DIA (6 *m/z* precursor specificity after demultiplexing) – available at https://panoramaweb.org/Mag-Net.url. The PRM survey assay resulted in similar reproducibility for those samples, with highly correlated abundances across QC samples (Fig. 6*B*).

Between individuals diagnosed as Parkinson’s and those that are not Parkinson’s, a total of 671 peptides mapping to 315 protein groups have significantly altered abundance (Fig. 6*C*). This includes peptides mapping to the proteins TERA (also known as VCP), CD36, RAB11 A/RAB11 B, G3P (aka GAPDH), and 1433E (aka YWHAE) which have previously been associated with Parkinson’s disease (44, 45, 46, 47, 48, 49, 50, 51) (Fig. 6*D*). Based on gene ontology enrichment analysis of the proteins with significantly changing peptides, there is an enrichment for proteins involved in Platelet Aggregation (GO:0070527) and Homotypic Cell-Cell Adhesion (GO:0034109).

## Discussion

Many diseases and disorders are still in dire need of robust and reliable assays that can monitor disease progression and/or assess the effectiveness of intervention. Biofluids remain an important reservoir for biomarker discovery and assay development. Two of the most important biofluids are blood plasma and cerebrospinal fluid as they provide a relatively non-invasive way to sample proteins for use as biomarkers that can aid in the detection, prognosis, and monitoring of the progression of disease. Here we demonstrate the development of highly multiplex targeted assays in both CSF and from enriched plasma EVs that were implemented in a few days of instrument time using a cost-effective quadrupole-radial ejection linear ion trap mass spectrometer – the Stellar (28).

We demonstrate the value of using DIA data collected using GPF to create a library that can inform the development of highly multiplex targeted peptide PRM assays in CSF and plasma. GPF is powerful because the method has a similar selectivity (*e.g.* similar quadrupole precursor isolation width) and sensitivity (based on the injection time) as fully targeted data acquisition. GPF normally comes at the expense of requiring multiple injections of the same sample to cover the *m/z* range of interest with sufficiently narrow DIA windows and long enough injection times (20, 26). However, use of this GPF DIA data enables assays to be developed directly within the sample matrix and the resulting library can be mined to create assays for different purposes (27). Furthermore, we make use of a new Skyline external tool, PRM Conductor, that can select peptides representative of proteins not only based on traditional mass spectrometry and chromatographic performance metrics but also based on the optimal use of the entire chromatographic separation (28).

We show that it is feasible to use libraries generated using GPF on either high-resolution accurate mass Orbitrap platforms or directly using the nominal mass resolution Stellar MS. The Adaptive RT alignment performs well between instrument platforms and with data generated in different laboratories. That said, while the library generated using the Orbitrap platform was significantly larger than the library generated using Stellar MS, the assay generated using the Stellar nominal mass GPF DIA data had better reproducibility at lower concentrations. This suggests that using an high-resolution accurate mass instrument to inform a targeted assay with a nominal mass resolution linear ion trap might not always be ideal. It is likely that peptides selected from Orbitrap data might not be as selective or have a similar dynamic range when transferred to the nominal mass resolution Stellar MS. Thus, selecting peptides using the GPF data on the Stellar MS will likely have greater success in a targeted PRM experiment using a Stellar MS.

There is a growing need for increasing the multiplex capacity of targeted quantitative protein assays. We explored the trade-offs of multiplex assays on the Stellar MS by testing two assays of different size – 3501 or 1599 precursors. The quantitative performance of the smaller assay was slightly better, with greater precision and lower limits of quantification. This improved quantitative performance is because of the reduced number of concurrent precursors, which enables longer ion injection time and an increase in the number of ions contributing to each peptide measurement (28, 52, 53, 54). The optimal size of an assay will vary based on the goals of the experiment and the chromatography set up, and selecting optimal target multiplexing is aided through the PRM conductor tool.

We demonstrate the use of GPF DIA to inform the development of highly multiplex PRM assays that either target known proteins of interest or target a broad selection of proteins that allow us to survey the proteome. In CSF we constructed an assay to measure 101 proteins of interest, targeting all peptides detected from these proteins using a GPF DIA analysis. While many peptides from the same protein change in the same direction between conditions, our ability to target many peptides per protein increases the chance of detecting individual peptides that change significantly between groups. We believe these large assays will be important in the validation or confirmation of many biologically interesting proteins in a single assay with minimal effort. With the large PRM assays in the Mag-Net enriched plasma EVs, we present a use case for a targeted assay that is not informed by a specific biological hypothesis. By selecting for reproducibly measured precursors while maximizing the number of precursors we target across a gradient and across the precursor range, we are still able to capture interesting biology in a dementia plasma cohort. We believe these highly multiplex quantitative assays that monitor biomedically relevant proteins will provide a much-needed bridge from discovery experiments to large scale clinical studies.

## Data Availability

Instrument raw files, database search results, and Skyline files are available on Panorama Public at https://panoramaweb.org/stellar-biofluid-prm.url and was assigned the ProteomeXchange ID PXD065471. The PRM conductor tool is available as a part of the Skyline External Tool store and can be installed from the Skyline UI itself or online from the Tool store at https://skyline.ms/tools.url. An additional tutorial for the use of PRM conductor is available through Panorama Public at https://panoramaweb.org/prm_conductor.url. Python and R code for statistical analysis and plotting are available on GitHub at https://github.com/uw-maccosslab/manuscript-stellar-biofluid.

## Supplemental data

This article contains supplemental data.

## Conflicts of interest

The authors declare no competing interests.

## Supplementary Data

Supplemental Tables

SupplementalTable_MNPlasma_PDvNonPD
